# Application of the Euro Clonality next‐generation sequencing‐based marker screening approach to detect immunoglobulin heavy chain rearrangements in mantle cell lymphoma patients: first data from the Fondazione Italiana Linfomi MCL0208 trial

**DOI:** 10.1111/bjh.17519

**Published:** 2021-05-18

**Authors:** Elisa Genuardi, Greta Romano, Marco Beccuti, Beatrice Alessandria, Donato Mannina, Catello Califano, Delia Rota Scalabrini, Sergio Cortelazzo, Marco Ladetto, Simone Ferrero, Raffaele A. Calogero, Francesca Cordero

**Affiliations:** ^1^ Department of Molecular Biotechnologies and Health Sciences ‐ Hematology Division University of Torino Torino Italy; ^2^ Department of Computer Sciences University of Torino Torino Italy; ^3^ IIGM ‐ Italian Institute for Genomic Medicine, c/o IRCCS Candiolo (Torino) Italy; ^4^ Candiolo Cancer Institute FPO ‐ IRCCS Candiolo Italy; ^5^ Azienda Ospedaliera Papardo‐ UOC di Ematologia Messina Italy; ^6^ U.O.C. Ematologia P.O. Pagani Salerno Italy; ^7^ Ematologia Fondazione del Piemonte per l’Oncologia – IRCCS Candiolo Italy; ^8^ Oncology Unit Clinica Humanitas/Gavazzeni Bergamo Italy; ^9^ Division of Hematology Azienda Ospedaliera SS Antonio e Biagio e Cesare Arrigo Alessandria Italy; ^10^ Hematology Division AOU “Città della Salute e della Scienza di Torino” Torino Italy; ^11^ Department of Molecular Biotechnology and Health Sciences University of Torino Torino Italy

**Keywords:** minimal residual disease, immunoglobulin genes, methodology, molecular biology, non‐Hodgkin lymphoma, polymerase chain reaction

## Abstract

Minimal residual disease (MRD) determined by classic polymerase chain reaction (PCR) methods is a powerful outcome predictor in mantle cell lymphoma (MCL). Nevertheless, some technical pitfalls can reduce the rate of of molecular markers. Therefore, we applied the EuroClonality‐NGS IGH (next‐generation sequencing immunoglobulin heavy chain) method (previously published in acute lymphoblastic leukaemia) to 20 MCL patients enrolled in an Italian phase III trial sponsored by Fondazione Italiana Linfomi. Results from this preliminary investigation show that EuroClonality‐NGS IGH method is feasible in the MCL context, detecting a molecular IGH target in 19/20 investigated cases, allowing MRD monitoring also in those patients lacking a molecular marker for classical screening approaches.

## Introduction

Monitoring minimal residual disease (MRD) in mantle cell lymphoma (MCL) is a powerful outcome predictor, validated in several clinical trials, independently of the therapeutic strategy.^1^, ^2^ Actually, the variable, diversity and joining (VDJ) clonal rearrangements of the immunoglobulin heavy chain (IGH) locus are the most common and standardized molecular targets in MCL. Overall, a reliable IGH‐based MRD marker is usually available in 70–75% of MCL patients. These markers are classically detected in diagnostic bone marrow (BM) and/or peripheral blood (PB) by polymerase chain reaction (PCR) and Sanger sequencing.^3^ They are fingerprints of the disease, trackable by sensitive and highly standardized allele‐specific oligonucleotide (ASO) PCR MRD approaches.^4^ Nevertheless, these approaches are labour‐intensive, time consuming and strongly dependent on tumour tissue infiltration. In fact, in case basal infiltration is <5%, marker identification could be problematic, since amplification of polyclonal signals might occur, eventually leading to misclassification of particular clonotypes.

As reported in several papers,^5^, ^6^, ^7^, ^8^ next‐generation sequencing (NGS) techniques could overcome these technical biases, reducing the time required for the experiment and increasing the number of patients screened in each experiment. Moreover, application of the (IGH)‐targeted NGS approach to B‐cell malignancies allows the amplification of a larger IGH repertoire with respect to the ASO‐PCR approach, identifying predominant clones, also called dominant clones, that are usually well distinguished from the polyclonal (healthy) IG background.^9^

Recently, Brüggemann *et al*.,^10^ in the context of the collaborative EuroClonality‐NGS Working Group, developed an IG/TR (T‐cell receptor) amplicon‐based NGS assay, multicentrically validated as a new approach to detect MRD targets in acute lymphoblastic leukaemia.

Based on these findings, we applied the EuroClonality‐NGS IGH method for marker screening in 20 MCL patients enrolled in the phase III “MCL0208” clinical trial (NCT02354313), sponsored by the Fondazione Italiana Linfomi (FIL). The experimental setting is defined following the standard operating procedures presented by Brüggemann *et al*., while NGS bioinformatics analysis was performed using HashClone, an easy‐to‐use and reliable bioinformatics tool implementing an efficient algorithm for clonality assessment and IGH‐based MRD assessment.^11^

## Methods

BM or PB samples were analysed by NGS and screened in parallel for IGHV rearrangements by PCR and Sanger sequencing of IGHV framework 1 and 2 regions (FR1–FR2),^12^ as scheduled in the trial. Moreover, polyclonal buffycoat (BC) and negative control (H_2_O) samples, were also included in the analysis. Libraries were prepared starting from 500 ng of genomic DNA and using the IGH FR1 multiplexed primers in the first amplification, while complementary Illumina‐specific barcode adaptors were added during the second amplification step. Libraries, after purification, were pooled and sequenced on the MiSeq Illumina platform (Illumina, San Diego, CA, USA). After assessment of the read quality by the FASTQC tool,^13^ HashClone was used for analysis of the sequenced samples. Among the B‐cell clonotypes identified, the dominant clone, i.e. the one with the highest abundance at the time of diagnosis, is defined by the following three‐step sequential procedure: (i) Filter A: selection of clones characterized by at least 80% sequence homology in each IGHV, IGHJ, and IGHD gene with respect to the sequences collected in the IMGT database,^14^ (ii) Filter B: selection of those clones associated with an abundance higher than 5% with respect to the total number of IGHV raw reads (as suggested by Faham *et al* ^15^), and (iii) selection of clones with an abundance higher than 1% of the remaining polyclonal background reads.

## Results

Twenty patients were included in our study: 10 BM and 10 PB samples, with a median tumour infiltration rate of 42·6% (range 1·7–82%), were screened for IGHV molecular markers by the NGS approach.

Out of the initial 3 836 clonal IGHV rearrangements, 188 were selected after the second filter step, with an average value of nine clonotypes per patient (Table I). In 19/20 samples a major IGHV clone was identified, while in one case (Patient ID 20) only a polyclonal background was observed (Table II).

**Table I:** Report of the number of reads mapping on the IGH junction, the number of clonotypes, the number of clonotypes passing Filter A, the number of clonotype passing Filter B and the presence (green check) or absence (red cross) of the dominant clone for each patient. [Colour table can be viewed at wileyonlinelibrary.com]

Patient ID	IGH junctional reads	Number of clonotypes	Clonotype passed Filter A	Clonotype passed Filter B	Dominant clone
1	12 419	164	40	1	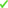
2	138 597	454	204	95	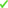
3	27 304	109	46	4	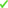
4	103 764	101	53	4	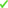
5	27 763	141	61	4	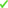
6	50 957	215	46	3	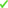
7	89 356	264	112	1	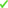
8	88 759	153	39	4	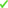
9	24 305	110	21	4	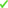
10	5478	191	21	3	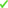
11	4080	115	58	3	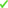
12	2119	111	52	3	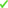
13	475	49	7	1	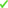
14	16 253	113	54	4	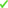
15	358 190	193	102	28	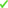
16	98 057	377	43	1	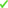
17	183 768	446	23	4	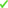
18	58 694	91	20	2	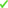
19	14 201	168	87	3	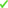
20	6596	271	50	16	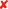

**Table II:**
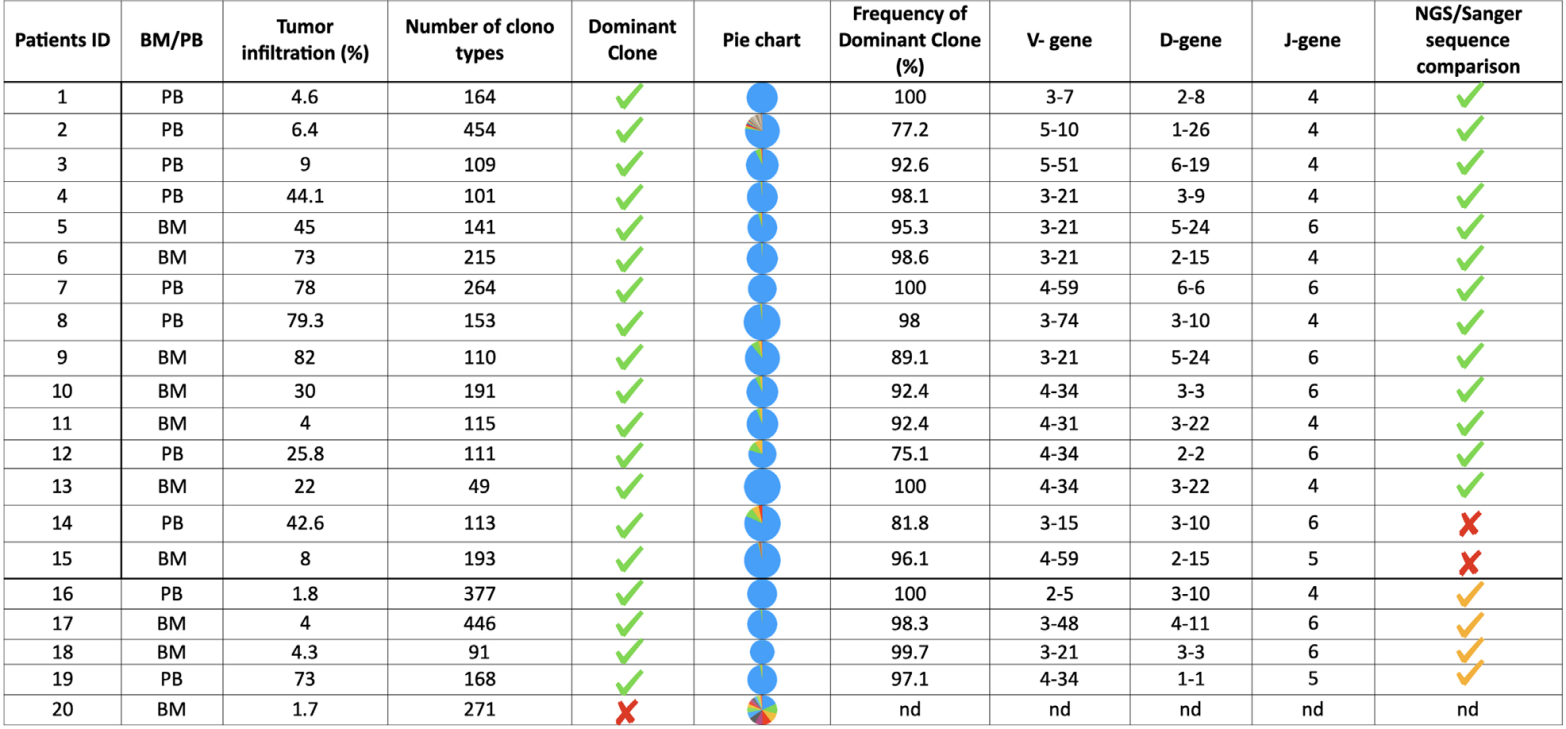
Comparison between next‐generation sequencing (NGS) and the classic polymerase chain reaction (PCR) marker screening approaches. For each patient d the type of sample on which the marker screening was implemented, the tumour infiltration value, the number of clonotypes selected by HashClone, the presence of the dominant clone, a pie chart showing the frequency values of the clones selected, the frequency of the dominant clone, the V, D and J gene of the major clone and the agreement between NGS and the Sanger technique are reported. Yellow check‐marks are used to mention the identification of the dominant clone in the NGS but not in the Sanger experiment. [Colour table can be viewed at wileyonlinelibrary.com]

Among the 19 IGHV NGS‐positive samples, 13 cases (68%) were fully concordant with the rearrangement identified by Sanger sequencing, two (11%) showed a different IGHV dominant clone compared to Sanger, while in four cases (21%) only, NGS succeeded to detect a clonal IGHV rearrangement. In one patient (Patient ID 20) neither NGS nor FR1–FR2 PCR/Sanger sequencing identified a clonal marker (Table II).

The aim of our study was to apply the amplicon‐based EuroClonality‐NGS marker screening approach to MCL patients. Even if the number of patients is limited, the high concordance between the dominant clones identified by NGS and those identified by Sanger sequencing (13/15, 87%) suggests that this approach is feasible and reproducible also in the MCL context. Overall, in the 13 cases where the NGS‐identified sequence was fully concordant with Sanger, MRD monitoring by ASO qPCR showed both high specificity and sensitivity levels in tracing the clone in the follow‐up samples. Moreover, in 2/15 cases, the implementation of a different bioinformatic algorithm, exploiting the HashClone computational suite^11^ provided an alternative IGHV dominant clone suitable for MRD analysis. Actually, ASO qPCR assay developed starting from the NGS clone showed a higher robustness than the previous one.

Finally, it is interesting to note that NGS showed better performance than the classic marker screening approach, allowing the identification of dominant clones also for four out of five patients in which the Sanger approach initially did not identify an IGH clonal rearrangement.

## Discussion

Based on our results, we implemented the NGS approach in laboratory routine because EuroClonality‐NGS allows marker identification in the majority of cases where Sanger sequencing failed. The few observed discordant cases between NGS and Sanger were validated as reliable markers for MRD by ASO qPCR. The performance of the EuroClonality‐NGS multiplexed two‐step FR1 PCR approach might be improved with the implementation of IGH‐FR2 and FR3 primer sets in the few cases in which both FR1‐NGS and Sanger failed.

In conclusion, our study demonstrates the feasibility of the EuroClonality‐NGS approach for MRD marker screening also in MCL patients and hints at the next steps to further improve its applicability.

## Conclusions

In our opinion, NGS‐based IGH marker screening might add to the current **armamentarium** of MRD methods to investigate the predictive value of tracking the major tumour clones detected by the high‐throughput approach in different therapeutic schedules.

This issue should be pursued by allowing a multi laboratory widespread use of this technology in the context of academic collaborations, with the final goal to drive the way towards a personalized treatment based on every patient specific biological features and risk profile.

## Author contributions

EG, GR, BA, MB SF and FC performed the research, analysed the data and wrote the paper. MB, SF, RAC and FC contributed essential reagents and tool. DM, CC, DRS, ML and SF contributed to clinical trial and patients enrolment.

## Conflicts of interest

The authors declare to have no potential conflicts of interest regarding the present work.
